# The importance of planning in the face of the COVID-19 pandemic in Paraguay

**Published:** 2020-09-01

**Authors:** Celeste Pavón de Miltos, Rainald Duerksen

**Affiliations:** 1 Director of Operations: Fundación Visión, Fernando de la Mora, Paraguay.; 2 Founder-Director: Fundación Visión, Paraguay and CBM Regional Inclusive Eye Health Advisor for Latin America.

**Planning, collective decision-making and care of staff members is at the heart of eye health provider Fundación Visión’s approach to COVID-19 in Paraguay.**

Fundación Visión is a large eye care provider in Paraguay which provides around 50% of the country’s ophthalmic services. There are four hospitals, which are managed in cooperation with public, private, national and international organisations. Our response to COVID-19 has been characterised by information and training, collective decision-making, operational and financial planning, and care of staff members.

## Information and training

On 4 March 2020, Paraguay issued a national plan in response to the pandemic. Based on this plan, Fundación Visión drew up an institutional protocol for COVID-19. The protocol included the definition of cases, internal and external communication plans, prevention and personal protective measures, triage of patients and employees, detection and management of suspected cases, infrastructure and care procedures, notification of cases, hospital hygiene, and the cleaning, disinfection, and sterilisation of ophthalmic materials and equipment.

**“In accordance with national guidelines, Fundación Visión had to stop providing all but emergency eye services for 90 days.”**

On 10 March, the day the second case of COVID-19 in the country was confirmed, we began to train staff members.

**From the field**

**Figure:**
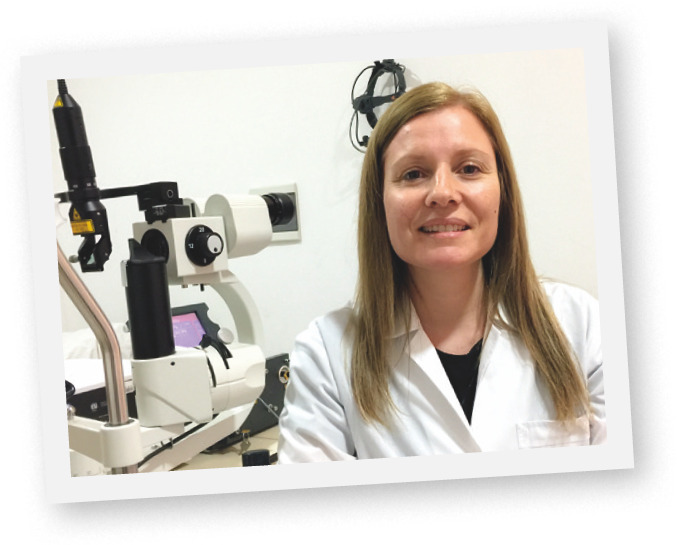

**Natalia Margonari**, an ophthalmologist and vitreoretinal specialist, is part of the Fundación Visión retinopathy of prematurity programme team. Dr Margonari and her team drive long distances every week to visit neonatal units at regional hospitals, where they screen premature babies for retinopathy of prematurity (ROP) and provide treatment, if needed. ROP can cause irreversible blindness unless it is detected early and managed appropriately. “We are in the midst of a pandemic that affects everyone worldwide, and we have been living in quarantine – but we cannot stop our work. We are taking strict protective measures so we can continue to reach premature and low birthweight babies who are at risk of ROP and do everything we can to save their vision.”

## Decision making and communication

One of the biggest challenges for Fundación Visión has been the lack of predictability inherent in the pandemic; as a result, decisions are made by consensus among the board, the council and the executive team.

A COVID-19 advisory team was created, with members from the Medical, Academic Coordination, Vision Program and Operations Directorates. The team issued regular newsletters, including about patient management, evidence-based personal protective equipment (PPE), and the measures needed for offices, study rooms, and operating rooms.

## Financial sustainability

In accordance with national guidelines, Fundación Visión had to stop providing all but emergency eye services for 90 days. Maintaining the same staffing levels and functionality during this time – with only minimal income – meant that the organisation would survive for only 90 days.

Therefore, in order to remain viable, we decided to reduce expenses by 80%, including suspending work for 94% of staff members for the 90-day period.

## Care of staff members

Fundación Visión continued to contribute towards social health insurance for staff members who had been suspended; a payment that was subsequently taken over – and expanded on – by the government.

An emergency committee, made up of representatives from each sector, meets often to discuss the need for monetary or other aid to employees and their families. This has included donation of non-perishable food kits. Pastors and volunteers are in permanent communication with staff members and their families to provide emotional and spiritual support in these uncertain times.

## Future plans

We have prepared a reactivation plan for restarting services, which will depend on the epidemiological situation and financial indicators.

**Figure:**
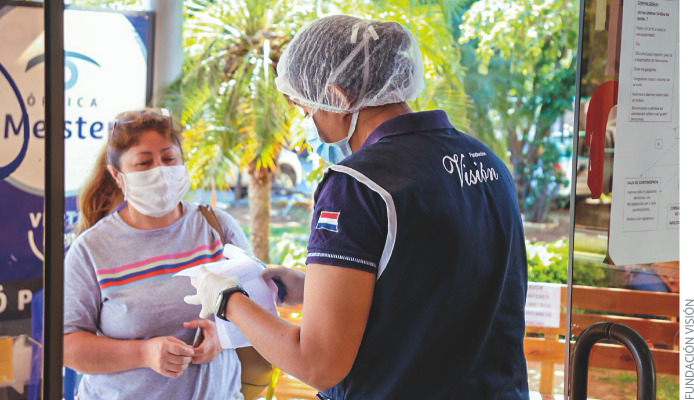
A health worker checks a patient’s appointment letter before allowing her to enter the hospital. **PARAGUAY**

